# Maternal Plasma miRNAs Expression in Preeclamptic Pregnancies

**DOI:** 10.1155/2013/970265

**Published:** 2013-09-09

**Authors:** Hailing Li, Qinyu Ge, Li Guo, Zuhong Lu

**Affiliations:** ^1^Department of Obstetrics and Gynecology, Zhongda Hospital, Southeast University, Dingjiaqiao 87, Nanjing 210009, China; ^2^State Key Laboratory of Bioelectronics, School of Biological Science and Medical Engineering, Southeast University, Sipailou 2, Nanjing 210096, China

## Abstract

*Objective*. Preeclampsia (PE) is a pregnancy-specific syndrome and one of the leading causes of maternal and fetal morbidity and mortality. The pathophysiological mechanisms of PE remain poorly known. Recently, circulating miRNAs are considered as potential useful noninvasive biomarkers. The aim of this study was to identify differentially expressed plasma miRNAs in preeclamptic pregnancies compared with normal pregnancies. *Methods*. Maternal plasma miRNA expression profiles were detected by SOLiD sequencing. Differential expressions between mPE/sPE and control group were found. Next, four differentially expressed plasma miRNAs were chosen to validate their expression in other large scale samples by real-time PCR. *Results*. In terms of sequencing results, we identified that 51 miRNAs were differentially expressed. Four differentially expressed plasma miRNAs (miR-141, miR-144, miR-221, and miR-29a) were selected to validate the sequencing results. RT-PCR data confirmed the reliability of sequencing results. The further statistical analysis showed that maternal plasma miR-141 and miR-29a are significantly overexpressed in mPE (*P* < 0.05). Maternal plasma miR-144 is significantly underexpressed in mPE and sPE (*P* < 0.05). *Conclusions*. Results showed that there were differentially expressed maternal plasma miRNAs in patients with preeclampsia. These plasma miRNAs might be used as notable biomarkers for diagnosis of preeclampsia.

## 1. Introduction

Preeclampsia (PE) is a pregnancy-specific syndrome characterized by hypertension (defined as systolic blood pressure *⩾*140 mmHg or diastolic blood pressure *⩾*90 mmHg) and proteinuria (300 mg or greater in a 24 h urine specimen and/or protein to creatinine ratio of >0.30) that occurs after 20 weeks of pregnancy [1]. Preeclampsia can lead to problems in the liver, kidneys, brain, and the clotting system and remained one of the leading causes of maternal and fetal morbidity and mortality [1]. It has been reported that PE complicates 3–8% of pregnancies [2]. Overall, 10% to 15% of direct maternal deaths are associated with preeclampsia and eclampsia. Risks for the baby include poor growth and prematurity, and perinatal mortality is high following preeclampsia [2]. The pathophysiological mechanisms of PE remain poorly known; most studies have implicated inadequate invasion of cytotrophoblasts into the uterine artery, leading to reduced uteroplacental perfusion pressure (RUPP) and placental ischemia/hypoxia, and an altered maternal immune response may play a role in the development of PE [3]. Many challenges remain regarding the prediction, prevention, and management of PE. Blood-based miRNAs may be potential biomarkers for early detection, diagnosis, and follow-up of this disease.

MicroRNAs (miRNAs) are a series of small (18–24 nt) endogenous noncoding single-stranded RNAs, which can regulate gene expression posttranscriptionally by a nonperfect pairing of 6–8 nucleotides with target mRNAs and subsequent formation of RISC complex resulting in degradation of target mRNAs [4, 5]. It is currently estimated that up to 30% of human genes may contain miRNAs' binding sites, which suggested a potential role of miRNAs as central regulators in the control of gene expression [6, 7]. Recent data indicate that miRNAs play a fundamental role in a variety of physiological and pathological processes in animals. They are believed to be critical in cell development, proliferation, communication [8], metabolism [5], stem-cell maintenance, and tissue differentiation [9]. MiRNAs have been implicated in a variety of human diseases, such as cardiovascular disease [10], primary muscular disorders [11], and cancer [12]. In addition, miRNAs have been reported involved in regulating pregnancy process [13]. There were studies focusing on placental miRNA expression in relation to preeclampsia, which showed that some special miRNAs may play a significant role in the development of preeclampsia [14–16].

Recently, a significant number of miRNAs have been observed outside of cells, including various body fluids. These miRNAs are stable and show distinct expression profiles among different fluid types [17–19]. The origin and especially the function of these circulating extracellular miRNAs remain poorly understood. A recent study reported that miRNAs could be selectively packaged into microvesicles and actively secreted. This suggested an interesting idea that extracellular miRNAs are used as mediators of cell-cell communication [19]. MiRNAs are stable in various bodily fluids; the sequences of most miRNAs are conserved among different species, and on expression of some miRNAs is specific to tissues or biological stages. Therefore, circulating miRNAs could be a potential independent predictive system for different diseases, compared with biomarkers derived from tissues [20]. The previous reports have shown the correlation between aberrant placenta miRNAs expression and preeclampsia, but only a few studies focused on circulating miRNA expression in preeclampsia.

Presently, high-throughput sequencing technology offers the possibility to obtain comprehensive and accurate measurement for transcripts. High-throughput sequencing is largely sequence independent, and it does not rely on the design of primers or probes specific to each miRNA. Since high-throughput sequencing provides the number of counts for each miRNA or transcript present in the sample, it is useful to quantitatively evaluate low expressing transcripts and has a wider dynamic range of quantification than microarray. The advancement of high-throughput sequencing technologies offers an unprecedented scale and depth of miRNA profiling, becoming a more available tool for researchers in studying microRNAs.

In this study we set out to investigate differentially expressed maternal plasma miRNAs in preeclamptic pregnancies, using genome-wide screening by Applied Biosystem's next generation sequencing system which is Sequencing by Oligonucleotide Ligation and Detection (SOLiD). These results may be the base for the further study to discover the pathophysiological mechanism and find preeclampsia-related biomarkers.

## 2. Materials and Methods

### 2.1. Participants

We divided the study into two steps: the first step was maternal plasma miRNA profiling; the second step was maternal plasma miRNA validation. We collected different patients for each step. In the first step, plasma samples were collected from 4 mild preeclampsia (mPE) cases, 4 severe preeclampsia (sPE) cases; and 4 normal pregnancies as control. Each group of samples were balanced mixed in a pool before sequencing. In the second step, plasma samples were collected from 16 mild preeclampsia cases, 22 severe preeclampsia cases; and 32 normal pregnancies as control. 

Preeclampsia was defined according to the International Society for the Study of Hypertension in Pregnancy; mPE was defined as maternal systolic blood pressure of *⩾*140 mmHg and/or diastolic blood pressure of *⩾*90 mmHg on 2 occasions separated by 6 hours and significant proteinuria (*⩾*300 mg of protein in a 24-hour urine specimen or *⩾*1+ by dipstick) after 20 weeks of gestation. sPE was defined as either severe hypertension (systolic blood pressure of *⩾*160 mmHg and/or diastolic blood pressure of *⩾*110 mmHg on at least 2 occasions 6 hours apart) plus mild proteinuria or mild hypertension plus severe proteinuria (*⩾*2 g/24 hr or *⩾*2+ by dipstick) [21]. The patients and controls were primiparas and well matched on age, gestational week, and gravidity. All blood samples were obtained from participants before treatment. Informed consents were obtained from all participants of this study. This project was approved by the Ethics Committee of Zhongda hospital, Southeast University. 

### 2.2. Samples Processing and RNA Extraction

The blood samples were collected in EDTA-containing tubes and then immediately centrifuged at 1600 ×g for 10 min to separated plasmas. Plasmas were carefully transferred into new tubes and stored at −70°C until used. Total RNA was extracted from plasma using mirVanaTM miRNA Isolation Kit (Ambion, Austin, TX, USA) following the manufacturer's instruction. The quantity and quality of obtained miRNA were measured with NanoDrop ND-1000 Spectrophotometer (NanoDrop, USA).

### 2.3. Maternal Plasma miRNA Profiling by SOLiD Sequencing

The small RNA libraries for SOLiD sequencing were prepared according to the manufacturer's protocol (Small RNA Expression Kit, Applied Biosystems). Small RNA samples were hybridized and ligated overnight with 5′ and 3′ adaptors, reverse transcribed, RNase H-treated, and PCR amplified. Then PCR products were cleaned up and selected on agarose gels by size 105–150 bp. Template bead preparation, emulsion PCR, and deposition were performed using the SOLiD V2 sequencing system (AppliedBiosystems) at the State Key Lab of BioelectronicsLaboratory, Southeast University of China. 

### 2.4. Sequencing Data Analysis

Mapping of SOLiD reads was analyzed by SOLiD System Small RNA Analysis Pipeline Tool (RNA2MAP, version 0.5.0). After filtrating the other human noncoding RNAs, including rRNA, tRNA, snRNA, and snoRNA, the remaining unique small RNAs were aligned with miRBase release 14.0 and the database of human genome to identify known miRNAs. Raw expression values (read counts) were obtained after these steps. Since low copy numbers were less reliable, those miRNAs with less than 10 copies were subtracted. To compare miRNA expression across datasets, the total copy number of each sample was normalized to 1.000.000. Fold change was calculated based on the normalized counts.

### 2.5. Real-Time PCR Validation

Real-time PCR (RT-PCR) analysis was performed on other groups of patients and controls. Complementary DNA (cDNA) was generated from 300 ng mirVana-enriched miRNA fractions in a 20 *μ*L reaction volume using the reverse transcription kit (Promega). MicroRNA primers were designed at the 5′ regions of the transcripts as to avoid potential mispriming due to the sequence variation observed at the 3′ ends of microRNA transcripts. Taqman probes were designed and synthesized for use in this study (Invitrogen, Shanghai). The primer and probe sequence could be seen in Table 1. Quantitative PCRs were performed with ABI PRISM 7500 real-time PCR system (Applied Biosystems); each sample was analyzed in duplicate, and the mean was used to determine miRNA levels. 

In the present study, relative quantification was used to analyze the expression of miRNA in different pregnant samples. The 2^-ΔΔCT^ method is a convenient way to analyze the relative changes in gene expression from real-time quantitative PCR experiment. Maternal plasma miRNA concentration was expressed as multiples of the median (MoM) for normal pregnancies.

### 2.6. Statistical Analysis

The characteristics of study subjects were compared using Mann-Whitney test. The significance of maternal plasma miRNA levels was determined by Mann-Whitney, Kruskal-Wallis test which was suitable. All *P* values were two sided, and less than 0.05 was considered statistically significant. All statistical calculations were performed by the SPSS software (version 18.0)

## 3. Results

### 3.1. Characteristics of Subjects

The characteristics of participants were described in Table 2. There were no significant differences of maternal age, gestational age, prepregnancy weight, and gravidity among the subjects and controls. All of pregnant women were primiparas. 

### 3.2. SOLiD Sequencing Results

**Figure 1:**
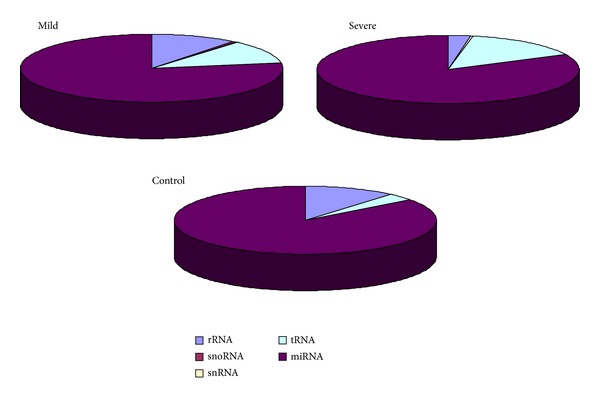
Distribution of different small RNA species in both groups and control.

Three groups of samples were used for preparation of small RNA libraries, mild preeclampsia group (*n* = 4); severe preeclampsia group (*n* = 4); normal pregnancy control group (*n* = 4). In addition, samples of each group were mixed into a pool. The resulting three RNA libraries were sequenced on the SOLiD/ABI platform. Raw sequencing results were mapped to miRBase release 14.0 and Human Genome RefSeq Hg19. Read counts were obtained by summing the number of reads that mapped uniquely to these reference databases. By this means, we obtained 15,499 reads in mild preeclampsia, 52,151 reads in serve preeclampsia, and 75,739 reads in normal pregnancy control, respectively. The distributions of small RNA species were shown in (Figure 1). It could be found that miRNA was the main component in maternal plasma small RNAs.

**Figure 2:**
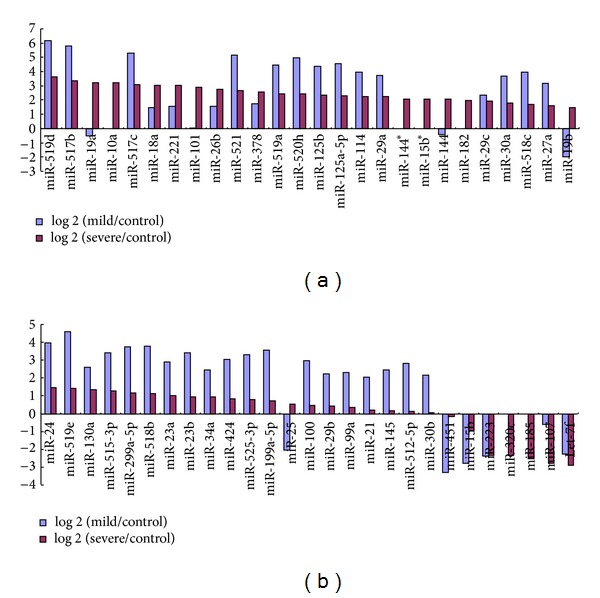
Differentially expressed maternal plasma miRNAs recieved by sequencing results, *y*-axis showed fold change of differential expression of miRNAs.

In terms of sequencing results, we identified that 51 miRNAs were differentially expressed, in which 22 miRNAs were upregulated and 5 miRNAs were downregulated in severe preeclamptic plasmas, and 33 miRNAs were upregulated and 6 miRNAs were downregulated in mild preeclamptic plasmas, when compared with normal pregnancy controls, respectively, (fold change >2 or <0.5 as the criterion for identifying differential expression) (Figure 2). 

### 3.3. PCR Validation of Differential Expression of Maternal Plasma miRNAs

**Figure 3:**
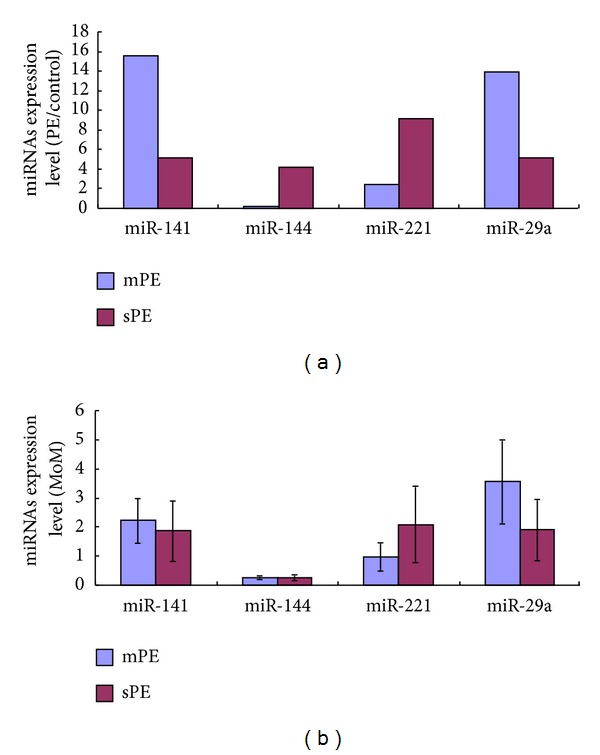
Real-time RT-PCR validation of maternal plasma miRNAs. (a) Differential expression results of maternal plasma miRNAs received by sequencing. (b) Differential expression results of maternal plasma miRNAs received by real-time RT-PCR.

We performed a real-time PCR utilizing 4 miRNAs (miR-141, miR-144, miR-221, and miR-29a) selected from those differentially expressed miRNAs in large scale samples to validate the sequencing results. In this step, maternal plasma miRNAs concentrations were expressed as multiples of the median (MoM) for normal pregnancies. As shown in Figure 3, the RT-PCR data conformed upregulation of miR-141, miR-221, miR-29a in sPE, miR-141, miR-29a in mPE and downregulation of miR-144 in mPE, in substantial agreement to our sequencing results. 

### 3.4. Expression of Maternal Plasma miRNAs in Preeclamptic Patients

**Figure 4:**
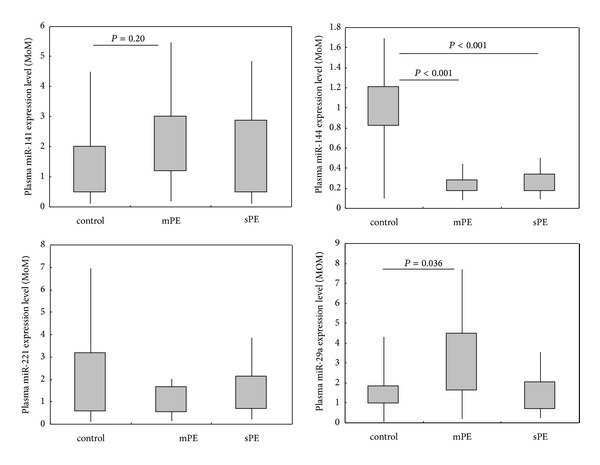
Maternal plasma miR-141, -144, -221, and -29a expression in pregnancies with or without preeclampsia.

We further analyzed the correlation between these plasma miRNAs expression level and the extent of preeclampsia. Statistical analysis (Figure 4) showed that maternal plasma miR-141 and miR-29a are significantly overexpressed in mPE when compare to normal control (*P* < 0.05), and maternal plasma miR-144 is significantly underexpressed in mPE and sPE when compared to normal control (*P* < 0.05). 

## 4. Discussion

At present, the role of miRNA in the pathogenesis of preeclampsia receives extensive attentions. The first research that linked miRNA and PE was conducted by Pineles et al. [14]. They investigated placental expression of 157 miRNAs among women with complicated pregnancies (preeclampsia and small for gestational age). They found that expression of miR-182 and miR-210 was significantly higher in PE than in the control group. The targets of both miR-182 and miR-210 are enriched in immune processes, which support the association between abnormal immune responses and preeclampsia. Zhu et al. [15] investigated placental miRNA expression of preeclampsia cases (mild and severe) and controls who had elective cesarean section with microarray. They reported that 34 miRNAs were differentially expressed (11 overexpressed and 23 downregulated) in preeclamptic placenta, notably in several miRNA clusters that include a region around 14q32.31 (a human-imprinted region) (miR-411, -377, and -154*). At about the same time, Hu et al. [16] conducted staged (screening microarray and validating qRT-PCR) investigations of placental miRNA expression and risk of severe preeclampsia. In their study, 27 miRNAs were differentially expressed (20 upregulated and 7 downregulated) among preeclamptic placenta. This research revealed that some angiogenic growth factors were potential targets of the altered miRNA, such as cysteine-rich 61 (CYR61), PlGF, and VEGF-A which were targets of miR-222, miR-335, and miR-195, respectively. Recently, Enquobahrie et al. [22] found that eight miRNAs were differentially expressed (miR-210 upregulated and miR-328, miR-584, miR-139-5p, miR-500, miR-1247, miR-34c-5p, and miR-1 downregulated) in placentas among preeclampsia cases compared with controls. These miRNAs target genes that participate in organ/system development (cardiovascular and reproductive systems), immunologic dysfunction, cell adhesion, cell cycle, and signaling. The association between preeclampsia and altered miRNA expression suggests the possibility of a functional role for miRNA in this disease. These differentially expressed miRNAs may play an important role in the pathogenesis of preeclampsia and may become diagnostic markers and therapeutic target for preeclampsia.

Recent studies reported that miRNAs could be selectively packaged into microvesicles and actively secreted, making them the potential candidates as mediators in cell-cell communication [19]. Therefore investigating differential circulating miRNAs expression may help us understand the molecular mechanism of maternal-fetal interaction in preeclampsia. Previous studies mainly focus on placental miRNA expression in relation to preeclampsia. Since few investigators have studied circulating miRNA associated with preeclampsia, we performed a comprehensive analysis of maternal plasma miRNA expression profile in preeclamptic pregnancies using a genome-wide SOLiD sequencing method. The sequencing results revealed that 51 miRNAs were differentially expressed, in which 22 miRNAs were upregulated and 5 miRNAs were downregulated in severe preeclamptic plasmas and 33 miRNAs were upregulated and 6 miRNAs were downregulated in mild preeclamptic plasmas, when compared with normal pregnancy controls, respectively. As shown in Figure 2, most of these differentially expressed plasma miRNAs indicate more significant dysregulation in mild preeclampsia group than in severe preeclampsia group. 

For miRNA measurement, it is well acknowledged that there is low correlation of results obtained from different platforms or even from the same platform using products from different vendors [23, 24]. For example, there were a few overlaps between those findings in placenta miRNAs associated with PE. The reliability of the results always has been questioned. The short length and high sequence similarities among some miRNAs likely contribute to the inconsistency of measurement results due to the problems in designing specific primers for RT-PCR or probes for microarrays. Additionally, sequencing of miRNAs has revealed significant sequence heterogeneity at the 3′ and 5′ ends (termed isomirs) that may further complicate measurements [25]. At present, RT-PCR is often considered a “gold standard” in the detection and quantitation of gene expression. We thus validated part of the sequencing results using RT-PCR to evaluate the reliability of results.

Preeclampsia is a systemic vascular endothelial disorder. The pathophysiological mechanisms of preeclampsia have been elusive, but some parts of the puzzle have begun to unravel. Genetic factors such as leptin gene polymorphism, environmental and dietary factors such as Ca^2+^ and vitamin D deficiency, and comorbidities such as obesity and diabetes may increase the susceptibility of pregnant women to develop preeclampsia. An altered maternal immune response may also play a role in the development of preeclampsia. According to some miRNAs' known functions, we chose four miRNAs (miR-141, miR-144, miR-221, and miR-29a) to test their plasma levels in pregnancies with or without preeclampsia. 

We regard miR-141 as a placenta specific miRNA. A prior report searched for placental miRNAs in maternal plasma. They analyzed miRNAs in the placenta, maternal blood cells, and plasma and found 17 candidate miRNAs, and the 4 miRNAs (miR-141, miR-149, miR-299-5p, and miR-135b) present at highest concentration in placentas were analyzed in maternal plasma by RT-PCR. The result showed that miR-141 was more stable in maternal plasma [26]. MiR-144 is chosen for its regulating function in ischemia and hypoxia. It is well known that the first step for the development of preeclampsia is the inadequate placental cytotrophoblast invasion, impaired trophoblast invasion, and inadequate maternal spiral artery remodeling which results in placental ischemia and hypoxia [27]. A previous study showed that miR-144/451 targeted CUGBP2 and upregulated COX-2-PGE2, a downstream singling effect of CUGBP2 [28], and COX-2 gene, which is known for its critical role in implantation and also for its abilities to promote inflammation and tumorigenesis [29]. As well, the role of COX-2 in ischemic heart disease and tissue response to hypoxia is still debated [30, 31]. MiRNA profiles revealed that miR-221 is enriched in endothelial cells [32, 33]. Among the highly expressed miRNAs in HUVEC, miR-221/222 exerts antiangiogenic effects. Transfection of endothelial cells with miR-221/222 inhibits tube formation, migration, and wound healing of endothelial cells in vitro [32]. Consistently, another study demonstrated the antiangiogenic function of miR-221/222 in endothelial cells [33]. The underlying mechanism involves the downregulation of the protein expression of the predicted target c-kit and the receptor for stem cell factor [32]. In haematopoietic progenitor cells, the miR-221/222 family also reduces c-kit expression and, as a functional consequence, cell proliferation [34]. MiR-221/222 overexpression also indirectly reduces the expression of the endothelial nitric oxide synthase (eNOS) in Dicer siRNA-transfected cells [33]. Substantial evidence indicates that nitric oxide (NO) production is elevated in normal pregnancy and that these increases appear to play an important role in the renal vasodilatation of pregnancy [35]. Study from laboratory indicated that chronic NO synthase inhibition in pregnant rats produces hypertension associated with peripheral and renal vasoconstriction, proteinuria, intrauterine growth restriction, and increased fetal morbidity [36]. Recent studies have suggested that miR-29 has complex functions in various diseases. Strong antifibrotic effects of miR-29s have been demonstrated in heart, kidney, and other organs [37]. Estrogen-induced miR-29 expressed in CCl(4)-induced mouse liver injury [38]. MiR-29a may behave as a tumor suppressor [39] or promoter [40, 41] in different tumors. In addition, the aberrant expression of miR-29a can be found in many nonmalignant diseases, including liver fibrosis [42], diabetes [43], and Alzheimer's disease [44]. 

We detected above four miRNAs expression level in maternal plasmas by RT-PCR and estimated the reliability of sequencing results. In this step, other large scale plasma samples were collected for validation study. The RT-PCR results confirmed upregulation of miR-141, miR-221, miR-29a in sPE, miR-141, and miR-29a in mPE and downregulation of miR-144 in mPE. These data agree with our sequencing results, making the latter more convincing. The further statistical analysis showed that maternal plasma miR-141 and miR-29a are significantly overexpressed in mPE, and miR-144 is significantly underexpressed in mPE and sPE when compared to normal control (*P* < 0.05). Differently from our expected results, it seems that dysregulation of plasma miRNAs is notable in mPE cases more than in sPE cases. Does this mean that plasma miRNAs variety maybe associated with the early pathological changes of preeclampsia? Further studies examining plasma miRNA expression patterns in early pregnancy stage with risk of preeclampsia could help us answer this question. The upexpression of miR-141 and miR-29a in mPE indicated that these miRNAs may be the potential biomarkers for early diagnosis of preeclampsia. In our results, significantly lower expression of plasmas miR-144 in mPE and sPE made miR-144 more interesting. Although the pathophysiology of PE remains undefined, placental ischemia/hypoxia is considered an important factor. The failure of trophoblast invasion results in reduced uteroplacental perfusion pressure (RUPP) and ischemic placenta, leading to PE and fetal IUGR. As we have discussed above, miR-144 may be an important regulator in ischemia and hypoxia. An early study has shown that miR-144/451 cluster confers protection against simulated ischemia/reperfusion-induced cardiomyocyte death via targeting CUGBP2-COX-2 pathway [28]. PGI_2_ is an antiplatelet aggregator and a vasodilator participating in pathogenesis of PE. PGI_2_ is produced from the metabolism of arachidonic acid by the cyclooxygenase (COX)-1 and COX-2. Plasma and urinary levels of 6-keto-prostaglandin F1*α* (PGF1*α*), a hydration product of PGI_2_, are lower during severe PE than in normal pregnancy [45]. Another study has shown that COX-2-derived prostaglandins (PGs) are critical to implantation [29]. These previous works suggested that miR-144 may participate in the regulation of placental ischemia in PE. Currently, the prevention and treatment of PE are hindered by the fact that the etiology remains unclear. If the action of miR-144 in PE can be further confirmed, it may be a potential target for prevention or treatment of the disease.

## 5. Conclusion

In summary, we have shown that differential maternal plasma miRNA expression is associated with preeclampsia. The real-time PCR data verified high-throughput sequencing results and indicated that high-throughput sequencing technology is reliable in miRNA profile detection. Further analysis showed that aberrant expressed plasma miRNA, associated with preeclampsia, may be the potential noninvasive biomarker for preeclampsia diagnosis and a potential targets for prevention or treatment of the disease. 

## Figures and Tables

**Table 1:** Characteristics of selected miRNAs.

miRNA	Primers/probes	DNA sequence (5′-3′)
hsa-mir-141	RT-primer	gtcgtatccagtgcagggtccgaggtattcgcactggatacgacccatct
	Forward	gctaacactgtctggtaaagat
	Probe	acgacccatctaatggtctg

hsa-mir-144	RT-primer	gtcgtatccagtgcagggtccgaggtattcgcactggatacgacagtaca
	Forward	gctacagtatagatgatgtact
	Probe	tacgacagtacaagtagata

hsa-mir-221	RT-primer	gtcgtatccagtgcagggtccgaggtattcgcactggatacgacagagtg
	Forward	tcgtgcatccttttagagtg
	Probe	cgacccaaagcagcataggtca

hsa-mir-29a	RT-primer	gtcgtatccagtgcagggtccgaggtattcgcactggatacgactaaccg
	Forward	gtagcaccatctgaaatcggtt
	Probe	ggatacgactaaccgatttca

	Universal reverse	gtgcagggtccgaggt

**Table 2:** Characteristics of study subjects.

Characteristics	SOLiD sequencing			RT-PCR validation		
	Mild preeclampsia (*n* = 4)	Severe preeclampsia (*n* = 4)	Normal control (*n* = 4)	Mild preeclampsia (*n* = 16)	Severe preeclampsia (*n* = 22)	Normal control (*n* = 32)
Maternal age (years)	29 (23–36)	34 (28–39)	28 (26–30)	31 (26–39)	33 (24–43)	29 (25–36)
Gestational age (weeks)	35.5 (34–37)	35 (32–38)	37 (36–38)	36.2 (32–38)	34.7 (28–37)	36.8 (34–38)
Gravidity	2 (1–3)	1.5 (1-2)	1.25 (1-2)	1.8 (1–4)	2.1 (1–3)	1.6 (1–3)
Prepregnancy weight (kg)	60 (50–68)	65 (60–70)	59 (55–62)	64 (48–72)	67 (52–77)	62 (55–74)
Birth weight (kg)	2.97 (2.6–3.2)	2.81 (2.4–3.4)	3.35 (3.1–3.6)	2.86 (2.4–3.4)	2.71 (2.1–3.6)	3.62 (2.6–4.1)
Systolic blood pressure (mmHg)	143 (140–148)	170 (165–180)	100 (90–110)	144 (140–152)	172 (160–200)	108 (90–120)
Diastolic blood pressure (mmHg)	92.5 (90–95)	115 (110–120)	70 (60–80)	96 (90–105)	114 (110–120)	76 (60–88)

Values expressed as median (range).
